# Amyloid Precursor Protein (APP) Regulates Gliogenesis and Neurogenesis of Human Neural Stem Cells by Several Signaling Pathways

**DOI:** 10.3390/ijms241612964

**Published:** 2023-08-19

**Authors:** Raquel Coronel, Adela Bernabeu-Zornoza, Charlotte Palmer, Rosa González-Sastre, Andreea Rosca, Patricia Mateos-Martínez, Victoria López-Alonso, Isabel Liste

**Affiliations:** 1 Unidad de Regeneración Neural, Unidad Funcional de Investigación de Enfermedades Crónicas, Instituto de Salud Carlos III, Majadahonda, 28220 Madrid, Spain; adela.bernabeu@gmail.com (A.B.-Z.); cpalmer248@gmail.com (C.P.); rosags111@hotmail.com (R.G.-S.); andreeastefaniar@gmail.com (A.R.); patriciamateos1996@gmail.com (P.M.-M.); 2 Departamento de Biología de Sistemas, Facultad de Medicina y Ciencias de la Salud, Universidad de Alcalá, Alcalá de Henares, 28871 Madrid, Spain; 3 Unidad de Biología Computacional, Unidad Funcional de Investigación de Enfermedades Crónicas, Instituto de Salud Carlos III, Majadahonda, 28220 Madrid, Spain; victorialopez@isciii.es

**Keywords:** amyloid precursor protein, neural stem cells, neurogenesis, gliogenesis, signaling pathways, RNA sequencing

## Abstract

Numerous studies have focused on the pathophysiological role of amyloid precursor protein (APP) because the proteolytic processing of APP to β-amyloid (Aβ) peptide is a central event in Alzheimer’s disease (AD). However, many authors consider that alterations in the physiological functions of APP are likely to play a key role in AD. Previous studies in our laboratory revealed that APP plays an important role in the differentiation of human neural stem cells (hNSCs), favoring glial differentiation (gliogenesis) and preventing their differentiation toward a neuronal phenotype (neurogenesis). In the present study, we have evaluated the effects of APP overexpression in hNSCs at a global gene level by a transcriptomic analysis using the massive RNA sequencing (RNA-seq) technology. Specifically, we have focused on differentially expressed genes that are related to neuronal and glial differentiation processes, as well as on groups of differentially expressed genes associated with different signaling pathways, in order to find a possible interaction between them and APP. Our data indicate a differential expression in genes related to Notch, Wnt, PI3K-AKT, and JAK-STAT signaling, among others. Knowledge of APP biological functions, as well as the possible signaling pathways that could be related to this protein, are essential to advance our understanding of AD.

## 1. Introduction

The pathological involvement of amyloid precursor protein (APP) in Alzheimer’s disease (AD) has been widely documented in the last 30 years due to its involvement in the generation of β-amyloid (Aβ) peptide [1]. Aβ peptide is one of the proteolytic derivatives of APP that accumulates and aggregates in the brain parenchyma and leads to the formation of senile plaques [2]. The appearance of these plaques in the brain is a main hallmark of AD [3]. On the other hand, the possible relationship between APP and Down syndrome (DS) has also been highlighted because the human *APP* gene is encoded on chromosome 21 and is one of the genes that appear overexpressed in DS of complete trisomy 21 [4]. For this reason, some authors defend that people with DS manifest early-onset AD and cognitive impairment due to this additional copy of the *APP* gene [5]. Although considerable progress has been made in understanding the pathogenic role of APP, the biological function of this protein is still not well known, and there is no clear consensus on it [6]. Therefore, a more detailed understanding of the function and activity of APP is necessary.

APP is a glycoprotein expressed ubiquitously in a wide variety of tissues, being especially abundant in the brain [7]. The APP expression is detected at early stages of nervous system development, as well as in the adult brain, suggesting that this protein plays a key and important role at various stages of life [8,9,10]. In the last years, APP has been described as a multimodal protein capable of regulating and modulating various biological processes, such as the proliferation, differentiation, and maturation of stem cells [11,12]; synaptogenesis, synaptic plasticity, and neurite outgrowth [13,14]; generation of new neurons (neurogenesis) and glial cells (gliogenesis) [15,16,17]; neuronal migration [18]; as wells as its participation in transcriptional regulation [19]. Furthermore, the protein structure of APP resembles a cell adhesion molecule or a membrane receptor, suggesting that its function is based on cell–cell interaction and/or activation of intracellular signaling pathways [6,20,21]. Recently, APP has been considered a conserved receptor of Wnt signaling (a conserved signal transduction pathway that regulates a large number of cellular processes) [22]. However, it is still difficult to determine if these effects are due to full-length APP or if, on the contrary, these functions are regulated by APP proteolytic derivatives.

Cleavage of full-length APP in cell membranes by several secretases gives rise to two canonical pathways of proteolytic processing: non-amyloidogenic and amyloidogenic pathways. In them, different proteolytic fragments are generated, including the soluble domains of APP (sAPPα and sAPPβ), Aβ peptide, and APP intracellular domain (AICD) [23]. The non-amyloidogenic pathway is characterized by the non-production of the Aβ peptide. This is due to the action of the α-secretase, which cleaves APP within the Aβ domain, preventing its formation and aggregation. In this case, the fragments sAPPα and p3 are released to the extracellular medium as processing products [24]. On the other hand, the amyloidogenic pathway is characterized by the production of Aβ peptide. Unlike the α-secretase enzyme, β-secretase does not cleave APP within the Aβ domain, and its formation is not prevented. In this case, the fragments sAPPβ and Aβ are released to the extracellular medium [24]. Notably, in both processing routes, after the action of the γ-secretase enzyme complex, the AICD fragment is released intracellularly as a product of processing [23,24].

Due to its multiple cleavage sites and the production of several fragments, APP has been attributed to numerous functions in the nervous system, making it difficult to understand its true function/s. The soluble domains of APP (mainly sAPPα), as well as the AICD intracellular fragment, have also been associated with neuroprotective functions and/or regulatory roles of cell proliferation, neurogenesis, and gliogenesis [17,19,25,26,27]. Even the Aβ peptide, in its monomeric form, has been proposed as a regulator of cognitive and synaptic functions and/or processes of neurogenesis, gliogenesis, and recovery from injury [28,29,30,31].

In our previous study, we aimed to identify the intrinsic effects of APP in the differentiation of human neural stem cells (hNSCs). Our results have shown that a transient increase in the expression of APP favored the differentiation of these hNSCs towards glial cells (gliogenesis) and, at the same time, it prevented the differentiation of these cells towards neurons (neurogenesis). In that case, we suggested the APP/AICD/GSK-3β system as a possible molecular mechanism involved in the observed effects [32]. However, given the multifunctionality of APP, we think that other molecular mechanisms could also be involved. 

In the present study, we have evaluated the effects of APP overexpression in hNSCs at a global gene level by a transcriptomic analysis using the massive RNA sequencing (RNA-seq) technology. Specifically, we have focused on differentially expressed genes that are related to neuronal and glial differentiation processes, as well as on groups of differentially expressed genes associated with different signaling pathways, in order to find a possible interaction between them and APP.

## 2. Results

In this study, we used hNS1 cells as a model of hNSCs. This cell line has been previously characterized [32,33,34]. 

**Figure 1:**
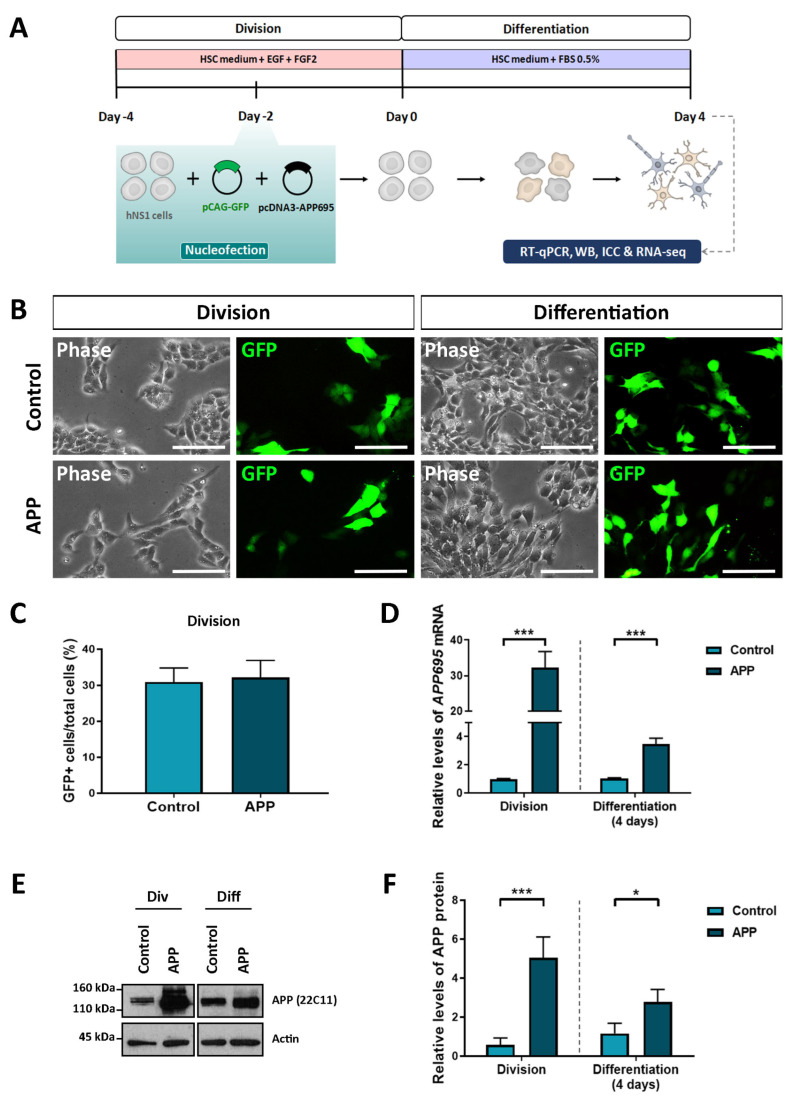
Confirmation of APP overexpression in hNS1 cells after transient nucleofection. (**A**) Schematic representation of the protocol used for proliferation (division) and differentiation (4 days after nucleofection) of hNS1 cells. (**B**) Representative images in phase contrast and the corresponding field with GFP+ cells in control-hNS1 cells and APP-hNS1 cells on different days after transfection (division and day 4 of differentiation). Scale bar = 50 μm. (**C**) Percentage of GFP+ cells with respect to total cells for both experimental groups (control and APP) in cell division. (**D**) Relative expression levels of *APP695* mRNA by quantitative real-time PCR (RT-qPCR) in APP-hNS1 cells and control-hNS1 cells on different days after transfection (division and day 4 of differentiation). (**E**) Western blot (WB) analysis of APP (using 22C11 antibody) in cell extracts of APP-hNS1 cells and control-hNS1 cells on different days after transfection (division and day 4 of differentiation). Actin was used as a loading control. (**F**) Relative protein levels of APP by densitometry analysis in both experimental groups (control and APP) on different days after transfection (division and day 4 of differentiation). Data represent mean ± SD (*n* = 3 for three independent samples). Statistical analysis was performed using a *t*-test between APP and control groups; * *p* < 0.05; *** *p* < 0.001.

In order to evaluate the effects of APP overexpression in hNS1 cells at the global gene level, we carry out a transcriptomic analysis using RNA-seq technology. For this, hNS1 cells were initially nucleofected with plasmids coding for APP695 and GFP (APP group) or empty plasmids and coding for GFP (control group). After transfection, hNS1 cells of both study groups were differentiated until day 4 (Figure 1A). Studies were done on a short time scale to avoid the likely compensatory mechanisms that could result from stable APP overexpression.

Prior to RNA-seq assays, we tested several parameters to confirm that APP overexpression had been achieved. In both groups, we verified that the cell transfection had been carried out correctly since we visualized GFP expression in the cell cultures (Figure 1B). Nucleofection efficiency obtained in both groups was equivalent (between 28 and 32% of GFP+ cells) (Figure 1C). In addition, quantitative real-time PCR analysis showed that relative levels of *APP695* mRNA are increased in hNS1 cells transfected with APP695-plasmid (APP group) against hNS1 cells transfected with empty-plasmid (control group), both in cell division and at 4 days of differentiation (Figure 1D). These results were confirmed at the protein level by Western blot analysis in both study groups (Figure 1E,F).

### 2.1. Transcriptome Analysis Reveals Gene Expression Differences in hNS1 Cells Due to APP Overexpression

Once the APP overexpression in our cells was confirmed, we performed RNA-seq experiments. Three independent replicates of APP-hNS1 cells and three independent replicates of control-hNS1 cells were processed for massive RNA sequencing.

**Figure 2:**
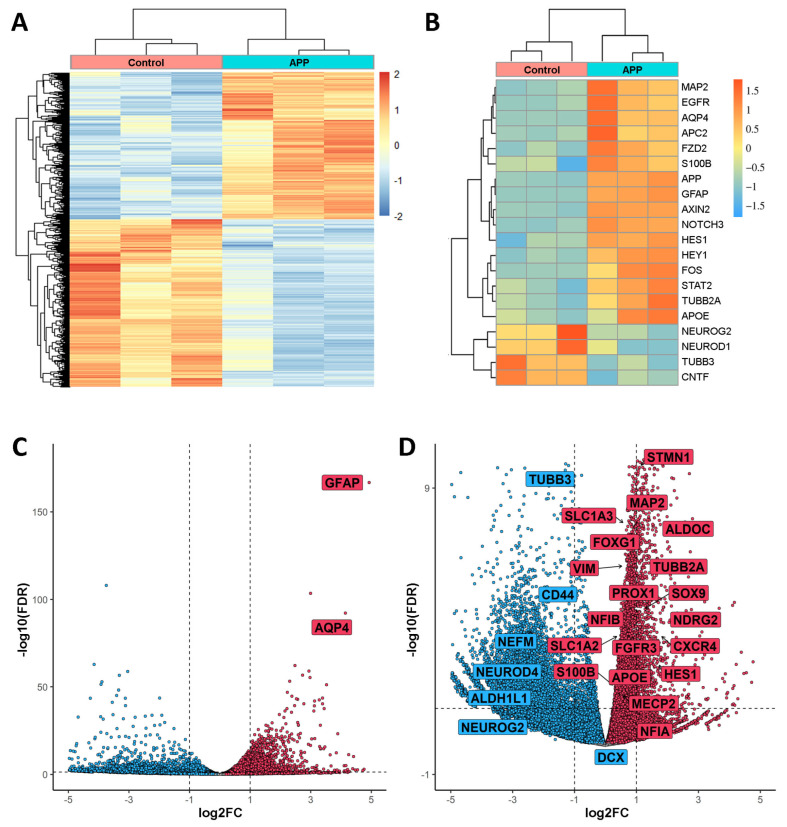
Representation of Differentially Expressed Genes (DEGs) found in APP-hNS1 cells versus control-hNS1 cells. (**A**) Heatmap showing differential RNA-seq counts of the genes with FDR ≤ 0.05 and sorted by logFold Change (logFC). There were three replicates of control samples (derived from control-hNS1 cells) and three replicates of APP samples (derived from APP-hNS1 cells). This heatmap was built using DESeq 2 on normalized gene read counts. (**B**) Heatmap of the three samples for each condition (APP-hNS1 cells and control-hNS1 cells) and gene expression levels of selected genes using average linkage clustering with Pearson correlation as the default distance metric. (**C**) Volcano plot from RNA-seq data of APP-hNS1 cells and control-hNS1 cells. The log2FC is plotted on the *x*-axis, and the negative log10(FDR) is plotted on the *y*-axis. Some relevant genes increased in APP-hNS1 cells (red dots) are indicated inside a box. (**D**) Replot of the volcano excluding the highest negative log10(FDR) genes. Red dots show the distribution of increased genes, and blue dots show the distribution of decreased genes in APP-hNS1 cells versus control-hNS1 cells. Some genes related to gliogenesis, neurogenesis, and different pathways mentioned in this study are indicated inside a box.

The analysis of the RNA-seq experiment found changes in gene expression due to overexpression of APP in hNS1 cells that, considering an FDR ≤ 0.05 as significant, resulted in 3032 genes upregulated and 2858 genes downregulated. The heatmap of Figure 2A showed a strong pairwise clustering of samples and a distribution of genes with significantly different expression patterns (upregulated and downregulated) (Figure 2A). 

In the RNAseq data, we found (as an example) that the increase of APP expression in hNS1 cells has a positive correlation with the increase of *APC2*, *APOE*, *AQP4*, *AXIN2*, *EGFR*, *FOS*, *FZD2*, *GFAP*, *HES1*, *HEY1*, *MAP2*, *NOTCH3*, *S100B*, *STAT2,* and *TUBB2A* expression. In addition, the increase of APP expression in hNS1 cells shows an inverse correlation with *CNTF*, *NEUROD1*, *NEUROG2,* and *TUBB3* expression (Figure 2B). 

The volcano plot remarks that, due to APP overexpression in hNS1 cells, two genes (*GFAP* and *AQP4*) exhibit the highest log2Fold Change (log2FC) and log10(FDR), indicating that both are two of the most upregulated genes as a consequence of APP overexpression (Figure 2C).

Similarly, we performed an enlargement of the volcano plot (by excluding the genes with the highest log10(FDR)) in order to visualize the distribution of some of the genes of interest. As an example, in the RNA-seq data, we found that the overexpression of APP in hNS1 cells is accompanied by an increase in the expression of *ALDOC*, *APOE*, *CXCR4*, *FGFR3*, *FOXG1*, *HES1*, *MAP2*, *MECP2*, *NDRG2*, *NFIA*, *NFIB*, *PROX1*, *S100B*, *SLC1A2*, *SLC1A3*, *SOX9*, *STMN1*, *TUBB2A*, and *VIM* genes; and a decrease in the expression of *ALDH1L1*, *CD44*, *DCX*, *NEFM*, *NEUROD4*, *NEUROG2,* and *TUBB3* genes (Figure 2D).

### 2.2. Functional Analysis of Differentially Expressed Genes (DEGs) in hNS1 Cells with APP Overexpression Shows Genes Involved in Neurogenesis and Gliogenesis

**Figure 3:**
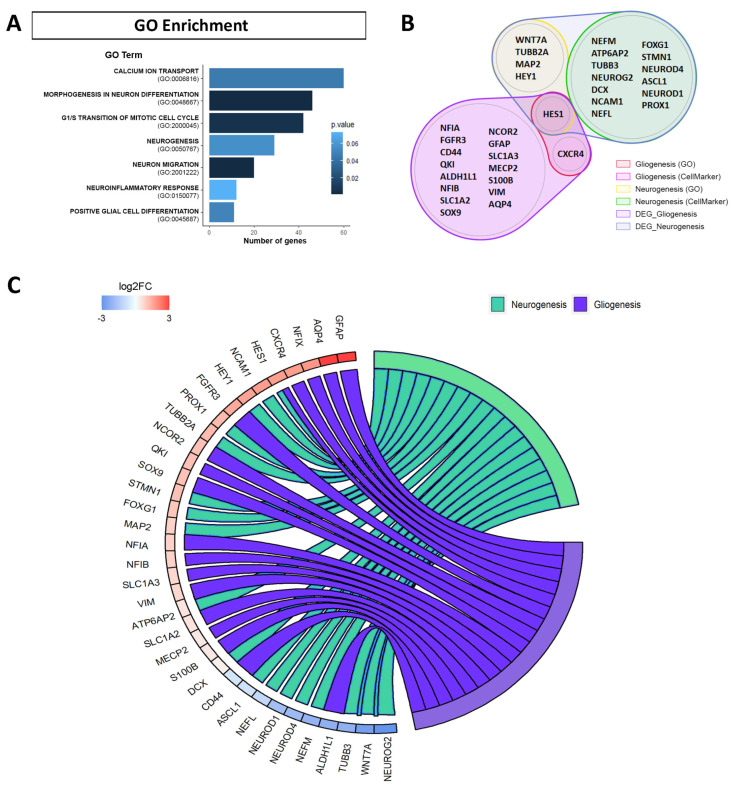
Functional enrichment of the genes differentially expressed (DEGs) in RNA-seq assays due to APP overexpression. (**A**) Gene Ontology (GO) enrichment analysis for the DEGs altered by APP overexpression versus control in hNS1 cells, obtained with Enrichr. Representation of the GO biological function terms is indicated with the bar length by the number of genes and the *p*-value by the color blue gradient. (**B**) Diagram with genes of RNA-seq assays dysregulated due to APP overexpression that are associated with gliogenesis and neurogenesis. Represented genes are based in terms of the GO, CellMarker, and Panglao databases. (**C**) Chord plot representing via colored ribbons the expression of genes associated with gliogenesis and neurogenesis. Genes are ordered according to log2FC in APP-hNS1 cells versus control-hNS1 cells, which are displayed in the intensity of red and blue squares next to the selected genes.

To obtain a deeper insight into the biological processes of upregulated and downregulated DEGs by APP overexpression, we performed Gene Ontology (GO) enrichment analysis. In this work, we focused on DEGs associated with biological process categories mainly related to neural stem cells (NSCs) in differentiation. A significant dysregulation was observed in the GO categories related to “calcium ion transport (GO:0006816)”, “morphogenesis in neuron differentiation (GO:0048667)”, “G1/S transition of mitotic cell cycle (GO:2000045)”, “neurogenesis (GO:0050767)”, “neuron migration (GO:2001222)”, “neuroinflammatory response (GO:0150077)” and “positive glial cell differentiation (GO:0045687)” (Figure 3A, Supplementary Table S1A).

We focused the analysis on the changes in the expression of DEGs related to cell differentiation in NSCs (gliogenesis and neurogenesis). Although GO is an international standardized gene functional classification system, we decided to include novel cell-type marker genes found in the CellMarker and Panglao databases (Figure 3B, Supplementary Table S1B).

After selection of the genes of interest, we show by chord plot the expression of upregulated and downregulated genes due to APP overexpression in hNS1 cells that are related with processes of gliogenesis (*ALDH1L1*, *ALDOC*, *AQP4*, *CD44*, *CNTF*, *CXCR4*, *FGFR3*, *GFAP*, *GJA1*, *HES1*, *MECP2*, *NCOR2*, *NDRG2*, *NFIA*, *NFIB*, *NFIX*, *QKI*, *S100B*, *SLC1A2*, *SLC1A3*, *SOX9*, *VIM*), and neurogenesis (*APOE*, *ASCL1*, *ATP6AP2*, *DCX*, *ENO2*, *FOXG1*, *HES1*, *HEY1*, *MAP2*, *NCAM1*, *NEFL*, *NEFM*, *NEUROD1*, *NEUROD4*, *NEUROG2*, *PROX1*, *SATB2*, *STMN1*, *TUBB2A*, *TUBB3*, *WNT5A*, *WNT7A*) (Figure 3C). 

In this plot, we observed that most of the genes selected and associated with the gliogenesis process had increased expression (*GFAP*, *AQP4*, *NFIX*, *CXCR4*, *FGFR3*, *NCOR2*, *QKI*, *SOX9*, *NFIA*, *NFIB*, *S100B*) in hNS1 cells with APP overexpression. In contrast, the genes selected and associated with the neurogenesis process showed an equivalent pattern between genes with increased expression (*NCAM1*, *HEY1*, *PROX1*, *TUBB2A*, *STMN1*, *FOXG1*, *MAP2*, *DCX*) and genes with decreased expression (*NEUROG2*, *WNT7A*, *TUBB3*, *NEFM*, *NEUROD4*, *NEUROD1*, *NEFL*).

These results indicate that APP overexpression favors the expression of gliogenic genes and some neurogenic genes in hNS1 cells while it decreases the expression of other neurogenic genes. Consequently, we think that the final effect of APP on neurogenesis in hNS1 cells, at day 4 of differentiation, depends on the balance of expression between upregulated and downregulated neurogenic genes.

### 2.3. Identification of Signaling Pathway Altered by APP Overexpression in hNS1 Cells

**Figure 4:**
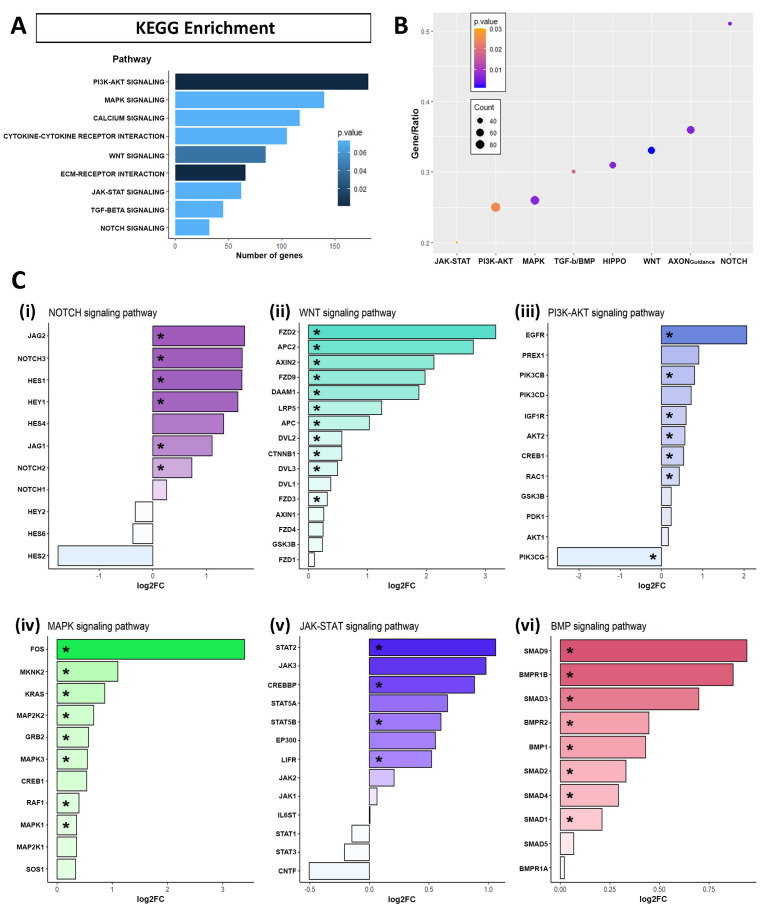
Signaling pathways enrichment of the genes differentially expressed (DEGs) in RNA-seq assays due to APP overexpression. (**A**) Kyoto Encyclopedia of Genes and Genomes (KEGG) pathways enrichment for all the DEGs altered by APP overexpression versus control in hNS1 cells, obtained with Enrichr. Representation of the signaling pathways are indicated with the bar length by the number of genes and the *p*-value by the color blue gradient. (**B**) Dotplot showing the results of KEGG pathways enrichment for the genes upregulated in APP versus control hNS1 cells. The *y*-axis represents the ratio between the number of genes participating in the analysis and the total annotated participants of the KEGG pathway. The dot color indicates the *p*-value, and the dot size indicates the number of genes. (**C**) Results of the RNA-seq analysis of APP-hNS1 cells versus control-hNS1 cells, representing the expression of genes as log2FC, included in KEGG signaling pathways of Notch signaling (**i**), Wnt signaling (**ii**), PI3K-AKT signaling (**iii**), MAPK signaling (**iv**), JAK-STAT signaling (**v**), and BMP signaling (**vi**). Significant differentially expressed genes are marked with an asterisk (*).

To obtain a deeper insight into the signaling pathways related to upregulated and downregulated DEGs by APP overexpression, we performed the Kyoto Encyclopedia of Genes and Genomes (KEGG) pathways enrichment analysis. When examining the pathways affected by APP overexpression in hNS1 cells, we found a significant dysregulation in the categories related to “cytokine-cytokine receptor interaction”, “calcium signaling pathway”, “MAPK signaling pathway”, “PI3K-AKT signaling pathway”, “NOTCH signaling pathway”, “TGF-BETA signaling pathway”, and “JAK-STAT signaling pathway” (Figure 4A, Supplementary Table S1C). These results were similar to those we obtained when performing the KEGG enrichment analysis only for the upregulated genes in the APP group (Figure 4B, Supplementary Table S1D). The results of KEGG enrichment analysis for the upregulated genes in the control group are presented in Supplementary Table S1E.

Moreover, we show by bar graph the expression of some genes selected and related to Notch signaling pathway (*HES1*, *HES2*, *HES4*, *HES6*, *HEY1*, *HEY2*, *JAG1*, *JAG2*, *NOTCH1*, *NOTCH2*, *NOTCH3*) (Figure 4(Ci)), Wnt signaling pathway (*APC*, *APC2*, *AXIN1*, *AXIN2*, *CTNNB1*, *DAAM1*, *DVL1*, *DVL2*, *DVL3*, *FZD1*, *FZD2*, *FZD3*, *FZD4*, *FZD9*, *GSK3B*, *LRP5*) (Figure 4(Cii)), PI3K-AKT signaling pathway (*GSK3B*, *AKT1*, *AKT2*, *EGFR*, *IGF1R*, *PDK1*, *PIK3CB*, *PIK3CD*, *PIK3CG*, *PREX1*, *RAC1*, *CREB1*) (Figure 4(Ciii)), MAPK signaling pathway (*CREB1*, *FOS*, *GRB2*, *KRAS*, *MAP2K1*, *MAP2K2*, *MAPK1*, *MAPK3*, *MKNK2*, *RAF1*, *SOS1*) (Figure 4(Civ)), JAK-STAT signaling pathway (*CNTF*, *CREBBP*, *EP300*, *IL6ST*, *JAK1*, *JAK2*, *JAK3*, *LIFR*, *STAT1*, *STAT2*, *STAT3*, *STAT5A*, *STAT5B*) (Figure 4(Cv)), and BMP signaling pathway (*BMP1*, *BMPR1A*, *BMPR1B*, *BMPR2*, *SMAD1*, *SMAD2*, *SMAD3*, *SMAD4*, *SMAD5*, *SMAD9*) (Figure 4(Cvi)). It should be noted that significant differentially expressed genes (*p*-value ≤ 0.05), in every way, are marked with an asterisk.

These results indicate that APP overexpression induces the expression of genes associated with Notch, Wnt, PI3K-AKT, MAPK, JAK-STAT, and BMP signaling pathways in hNS1 cells. Consequently, we think that these signaling pathways could be involved, at the molecular level, in the previously observed cellular effects (on gliogenesis and neurogenesis) in hNS1 cells.

### 2.4. Validation of Transcriptomic Results by Computational and Experimental Analysis

To investigate the interactions between the protein products of differentially expressed genes related to neurogenesis and gliogenesis, as well as the interactions between the protein products of differentially expressed genes related to several signaling pathways, we constructed a node network using the STRING database. In addition, in both cases, we studied the relationship with APP.

**Figure 5:**
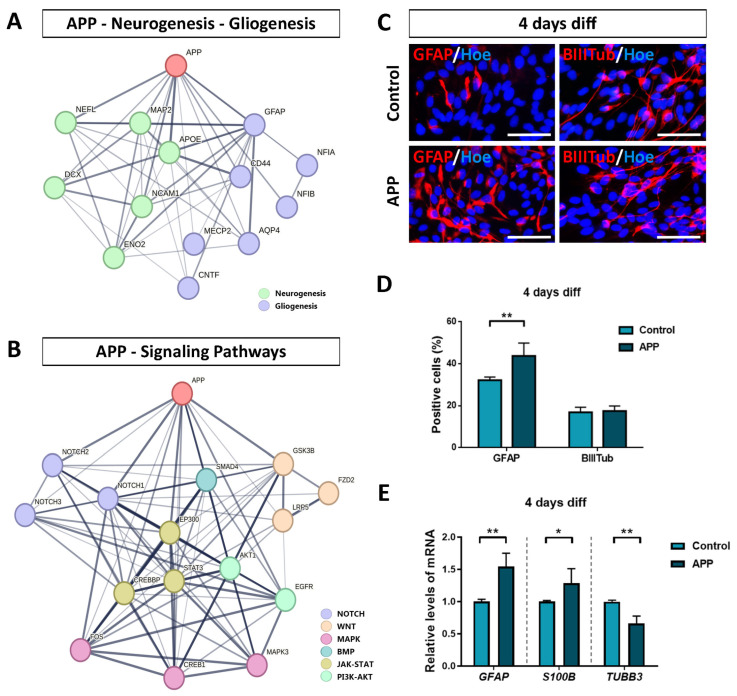
Validation of RNA-seq results on gliogenesis and neurogenesis processes in APP overexpression hNS1 cells. (**A**) STRING network of protein–protein interactions between APP, neurogenesis-related genes, and gliogenesis-related genes according to our transcriptomic analysis. (**B**) STRING network of protein–protein interactions between APP and signaling pathways-related genes according to our transcriptomic analysis. Thicker network edges show greater evidence of a biological relationship between the nodes (genes). (**C**) Representative images at day 4 of differentiation by immunocytochemistry (ICC), showing immunoreactivity for GFAP (red) and β-III-tubulin (BIIITub; red) in control-hNS1 cells and APP-hNS1 cells. Scale bar = 50 μm. (**D**) Percentage of positively stained cells for GFAP and β-III-tubulin markers relative to the total cells (Hoechst; blue) at day 4 of differentiation after nucleofection. (**E**) Relative expression levels of *GFAP*, *S100B,* and *TUBB3* mRNA by quantitative real-time PCR (RT-qPCR) in APP-hNS1 cells and control-hNS1 cells at day 4 of differentiation after nucleofection. Data represent mean ± SD (*n* = 3 for three independent samples). Statistical analysis was performed using T-test between APP and control groups; * *p* < 0.05; ** *p* < 0.01.

Computational analysis showed a high association between *APP* and neurogenic genes (*NCAM1*, *NEFL*, *APOE*, *MAP2*), as well as between *APP* and gliogenic genes (*GFAP*, *NFIB*, *CD44*) (Figure 5A). It should be noted that an existing relationship between *APP* and *DCX* or *AQP4* has also been described, although lighter than the previous ones, according to the STRING database. On the other hand, the computational analysis regarding signaling pathways revealed a high association between *APP* and *STAT3*, *EP300* (JAK-STAT signaling), *APP* and *MAPK3*, *CREB1* (MAPK signaling), *APP* and *NOTCH1*, *NOTCH2* (Notch signaling), *APP* and *SMAD4* (BMP signaling), *APP* and *AKT1*, *EGFR* (PI3K-AKT signaling), and *APP* and *GSK3B* (Wnt signaling and/or PI3K-AKT signaling) (Figure 5B). 

Altogether, these data indicate the possible interactions between APP and proteins associated with DEGs obtained by RNA-seq analysis (in relation to the biological processes of neurogenesis/gliogenesis and the different signaling pathways), which provides consistency to the conclusions presented above.

With the purpose of validating some RNA-seq results obtained (relating to gliogenesis and neurogenesis processes in APP overexpression hNS1 cells), we carried out immunocytochemistry assays to detect GFAP (glial cell marker) and β-III-tubulin (neuron marker) in both groups at 4 days of differentiation (Figure 5C). The data obtained showed that APP overexpression cells had a higher percentage of positive cells for GFAP (43.7 ± 6.2%) compared to the control group (32.2 ± 1.6%) (*p* < 0.01; *n* = 3) (Figure 5D). These results were further confirmed at the level of mRNA expression by quantitative real-time PCR analysis for *GFAP* and *S100B* (Figure 5E). On the contrary, the analysis of neuronal generation by immunocytochemistry experiments did not show statistically significant differences between percentages of positive cells for β-III-tubulin in the APP group (17.5 ± 2.3%) and control group (16.9 ± 2.4%) (Figure 5D). However, at the level of mRNA, we detected a significant decrease in the expression of *TUBB3* (Figure 5E), similar to the data obtained by RNA-seq.

Altogether, our results indicate that increased expression of APP in hNS1 cells promotes their differentiation toward a glial phenotype at the cellular and molecular level, increasing the expression of gliogenic genes. In addition, the high levels of APP expression also affect to neurogenesis process in hNS1 cells, although these changes are more heterogeneous at the general cell culture level, probably due to a dual role of APP (and/or its proteolytic derivatives) in the generation of new neurons.

## 3. Discussion

In previous work, we observed that a transient increase in the expression of APP at the intrinsic level favored the gliogenesis process and, at the same time, it prevented the neurogenesis process in hNSCs (hNS1 cell line). In that case, we suggested the APP/AICD/GSK-3β system as a possible molecular mechanism involved in these effects [32]. However, we think that other molecular mechanisms could also be involved. 

In order to obtain a global vision of the role played by APP in hNSCs, in this study, we performed a massive sequencing transcriptomic analysis using RNA-seq technology from hNS1 cells with APP overexpression.

After functional enrichment analysis (GO terms) of DEGs in hNS1 cells, we observed that transient APP overexpression promotes the expression of gliogenic genes and regulates the expression balance of neurogenic genes (some of them were found to be upregulated and others downregulated). These data suggest that APP, both at the intrinsic and extrinsic level, favors hNSCs gliogenesis, while its effects on neurogenesis seem to be variable according to its intrinsic/extrinsic analysis.

As described in many studies, cell proliferation and differentiation of NSCs are exquisitely controlled by intrinsic and extrinsic factors [35,36,37]. In addition, there is evidence that shows a differential influence of APP on the processes of proliferation and phenotypic specification of NSCs, depending on whether the effects are mediated by their extracellular proteolytic derivatives (sAPPα, sAPPβ and/or Aβ peptide, which would act as extrinsic factors) or by its intracellular proteolytic derivative AICD (which would act as an intrinsic factor) [16]. Thus, several studies affirm that sAPPα promotes neurogenesis (both in vitro and in vivo) in NSCs [11,38], whereas AICD appears to negatively modulate the process of neurogenesis in hNSCs [19].

Based on this, it is important to mention that we detected the presence of sAPPα in the extracellular media of hNS1 cells with transient APP overexpression by Western blot analysis. Therefore, we think it could be plausible that certain observed effects in this study (on cell fate specification or cell signaling activation) could be mediated by the sAPPα fragment in addition to full-length APP.

Likewise, previous studies from our group show that Aβ40 peptide (in its soluble and monomeric form) induces neurogenesis in hNSCs (hNS1 cell line) [29], while Aβ42 peptide (also in its soluble and monomeric form) is capable of promoting the processes of cell proliferation and gliogenesis [28]. Moreover, APP itself (in its full length) and its proteolytic derivatives interact with a wide variety of receptors and cell adhesion molecules [39,40,41], which in turn induce several signaling pathways, exponentially expanding the possibilities of study. For these reasons, we think that the final effect of APP on the phenotypic specification of hNSCs depends on the balance of their intrinsic and extrinsic functions.

Recently, growing evidence has shown that the amyloid assemblies function as regulators of several cell signaling pathways [42,43]. In this context, there could be the possibility that aggregated forms of Aβ peptide (as oligomers and fibrils) could mediate some of the observed cellular effects. In previous studies, we demonstrated that the Aβ42 peptide, in its oligomeric and fibrillar state, affects the phenotypic specification of hNSCs [44]. However, in the current work, aggregated forms of Aβ peptide have not been detected, although we cannot exclude the possible effects of soluble Aβ peptides.

In order to delve into the possible signaling pathways involved in the cellular effects observed (increase in gliogenic genes and balance of neurogenic genes), we also performed an enrichment analysis (KEGG pathways) of DEGs in hNS1 cells. Our results showed that transient APP overexpression promotes the expression of genes associated with Notch, Wnt, PI3K-AKT, MAPK, JAK-STAT, and BMP signaling pathways in hNS1 cells.

There are numerous studies linking Notch signaling [45], Wnt signaling [46], PI3K-AKT signaling [47], JAK-STAT signaling [48], and BMP signaling [49] with proliferation and phenotypic specification of NSCs. The involvement of some of these signaling pathways in certain cellular processes mediated by APP and/or its proteolytic derivatives has even been described. Specifically, it has been reported that sAPPα induces glial differentiation of NSCs through the Notch pathway [50], the JAK-STAT pathway [51], and the BMP pathway [17].

In relation to the Notch signaling pathway, several works support that Notch plays a key role in determining the cell fate of NSCs and has been described as a potent molecular switch [52]. There is accumulated evidence that demonstrates the participation of the Notch pathway in the differentiation of NSCs, promoting gliogenesis while preventing neurogenesis [53]. In addition, similar to APP, the Notch receptor can be cleaved by γ-secretase, generating the NICD fragment (Notch intracellular C-terminal domain), which regulates the transcription of target genes (like AICD fragment) [54].

Likewise, recent studies indicate that activation of the Wnt signaling pathway favors the neurogenesis of NSCs, while its inhibition leads to an increase in glial differentiation [55]. Moreover, increasingly authors defend a clear association between APP and Wnt pathway since the participation of APP in practically all segments of the Wnt signaling cascade has been determined. That is, at the ligand level (APP physically interacts with several Wnt co-receptors) [56], at the receptor level (APP has been described as a conserved Wnt receptor) [22], at the cytoplasmic level (APP and its proteolytic derivatives have been related to GSK-3β) [57], and at the nuclear level (APP forms a protein complex with β-catenin and prevents its translocation to the nucleus) [58].

**Figure 6:**
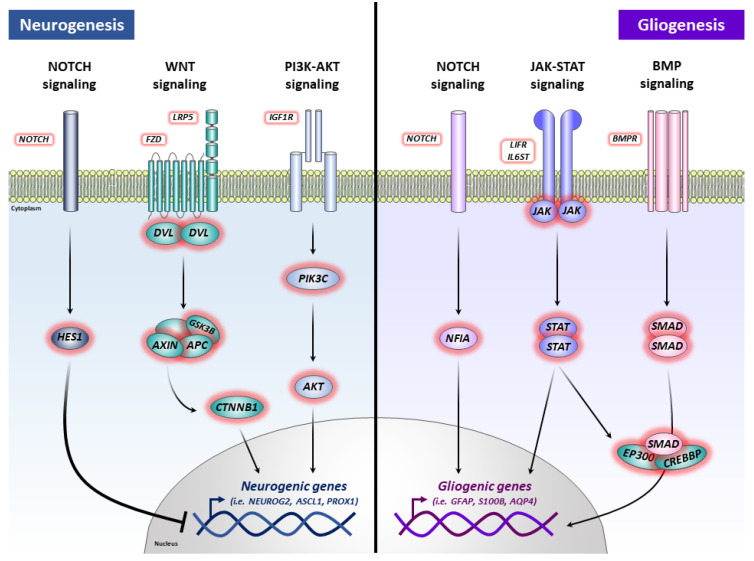
Schematic illustration of the suggested effects of APP overexpression on hNSCs. The positive and negative effects of APP on neurogenesis could be mediated, at a global level, by Notch signaling, Wnt signaling, and/or PI3K-AKT signaling. The positive effects of APP on gliogenesis could be mediated, at a global level, by Notch signaling, JAK-STAT signaling, and/or BMP signaling.

In conclusion, our data indicate a role of APP in controlling the phenotypic specification of hNSCs (an increase of gliogenic genes and balance of neurogenic genes), and these effects could be mediated through several signaling pathways (Notch, Wnt, PI3K-AKT, MAPK, JAK-STAT, and/or BMP) (Figure 6). This finding may contribute to the knowledge of APP physiological functions and elucidate the multiple roles of this protein and its proteolytic derivatives, being essential and necessary to be able to advance our understanding of AD pathogenesis.

## 4. Materials and Methods

### 4.1. Ethics Statement

The original human fetal tissues were donated to the research after signing an informed consent by the woman who suffered an abortion. The tissues were obtained and used in accordance with the Declaration of Helsinki of the World Medical Assembly (WMA) and the ethical standards of the Network of European CNS Transplantation and Restoration (NECTAR). Approval to use these tissues for research was granted by the University of Lund Hospital Ethics Committee, and its use was carried out in accordance with Spanish Law 14/2007 on Biomedical Research. More information about ethical statements of used cells origin in this study (hNS1 cell line) can be found in the original reports [33,34].

### 4.2. Cell Culture and APP Overexpression

We used hNS1 cells, a model of hNSCs with the capacity to differentiate into neurons, astrocytes, and oligodendrocytes. These cells are derived from the forebrain of a 9.5-week gestational age human fetus, which were immortalized with v-myc by retroviral infection. hNS1 cell line has been previously characterized [32,33,34].

In this study, hNS1 cells were cultured adherently on plates pre-treated with poly-L-lysine (10 μg/mL, Sigma, Merck, Darmstadt, Germany) and proliferated on a chemically defined human stem cell (HSC) medium supplemented with fibroblast growth factor (FGF2) (20 ng/mL, Peprotech, London, UK) and epidermal growth factor (EGF) (20 ng/mL, Peprotech). Proliferating hNS1 cells at 70–80% confluence were dissociated with trypsin (0.25%, Gibco, ThermoFisher Scientific, Waltham, MA, USA), centrifuged (4 min at 900 rpm), and nucleofected (Nucleofector Amaxa 2B, Lonza, Basilea, Switzerland) according to the manufacturer’s instruction. Briefly, for the APP overexpression group, hNS1 cells were pre-mixed with 4 µg of pcDNA3-APP695 plasmid (contains the human gene of APP695 isoform and was kindly provided by Dr. Daniel Lu, UCLA) in 100 µL of nucleofection solution (Lonza). Similarly, for the control group, hNS1 cells were pre-mixed with 4 µg of empty pcDNA3 plasmid (Invitrogen, ThermoFisher Scientific, Waltham, MA, USA) in 100 µL of nucleofection solution. In addition, in both study groups, hNS1 cells were co-transfected with 4 μg of the pCAG-GFP plasmid (Addgene, Teddington, UK) to track transfected cells. The program used for the nucleofection of hNS1 cells was A-031 in all cases. 

After transfection (day-2), hNS1 cells were plated in a proliferation medium, and after two days (day 0), they were differentiated on an HSC medium (without growth factors) and heat-inactivated fetal bovine serum (FBS) (0.5%, Gibco). We studied the effects of APP overexpression on a short time scale (4 days after nucleofection). In all cases, hNS1 cells were maintained in an incubator at 37 °C and 5% CO_2_ with relative humidity.

### 4.3. RNA Isolation

hNS1 cells cultivated in multiwell plates were dissociated with trypsin and centrifuged in PBS. Total RNA from pellets was isolated using a spin column kit (Rneasy Mini Kit, Qiagen, Hilden, Germany) according to the manufacturer’s instruction and treated with DNAses to avoid amplification of undesired genomic and plasmid DNA. 

### 4.4. Library Preparation and Transcriptomic Sequencing

For massive RNA sequencing (RNA-seq) assay, 2 µg of total RNA was isolated from three independent samples (*n* = 3) in each study group (at 4 days after nucleofection). All samples had high intact and quality RNA showing an RNA integrity number higher than 7.0. Samples were subjected to rRNA removal using a magnetic bead kit (Ribo-Zero Gold rRNA Removal Kit, Illumina, San Diego, CA, USA) according to the manufacturer’s instructions. Subsequently, the rRNA-depleted samples were purified, and the RNA-seq libraries were prepared using ScriptSeq v2 RNA-Seq Library Preparation Kit (Illumina, Inc., San Diego, CA, USA). RNA and library quality assurance were conducted using the Agilent 2100 Bioanalyzer System (Agilent Technologies, Palo Alto, CA, USA). The library was sequenced in single-end mode (75 bp) of an Illumina NextSeq 500 platform in the Genomics Unit of the Instituto de Salud Carlos III (ISCIII), obtaining an average yield of 40 million reads per sample.

### 4.5. Quality Control, Sequence Reads Alignment, and Analysis of Differentially Expressed Genes

The analysis started with the quality control of the RNA-seq raw sequences with FastQC [59] and adapter and quality trimming with Trimmomatic [60]. The filtered reads were aligned to the ENSEMBL human reference genome GRCh38.p13 (Genome Reference Consortium Human Build 38) using the STAR read aligner [61]. Afterward, the fragments per kilobase million mapped reads (FPKM) were calculated based on the read counts derived for each gene using the HT-Seq software v.0.6.1 [62]. Differential expression analysis was performed using the R package DESeq2 v.1.30.0 [63] that calculates the *p*-value by Wald’s test and correction of the multiple testing by the Benjamini–Hochberg method to achieve the adjusted *p*-value or False Discovery Rate (FDR). In this study, we considered as the differentiated expression genes (DEGs) the analyzed genes of FDR ≤ 0.05 and those with log2(Fold Change(FC)) > 0 and log2(FC) < 0 as upregulated and downregulated genes in APP-hNS1 cells versus control-hNS1 cells, respectively. This was represented in Heatmaps and Volcano plots generated using heatmap and the ggplot2 packages in R.

Transcriptomic data were deposited in NCBI Gene Expression Omnibus (GEO) and can be retrieved using the GEO series accession number: GSE233651.

### 4.6. Functional Annotation and Enrichment of the DEGs

We analyzed the RNA-seq genes altered by APP overexpression with the Enrichr web portal [64]. DEGs were annotated by the Gene Ontology (GO) for biological processes [65], the Kyoto Encyclopedia of Genes and Genomes (KEGG) for pathways [66], and CellMarker [67] and Panglao Database [68] for cell type markers. The GO and KEGG terms, with a *p*-value ≤ 0.05, were considered significant. Enriched terms related to our study are presented with bar and dot plot graphs done with the ggplot2 library in R.

The expression of diverse genes associated with neurogenesis and gliogenesis terms in GO, CellMarker, and Panglao Database are presented with a chord graph done with the GOChord library in R.

To investigate the protein-protein interactions network between APP and the protein products of genes differentially expressed in this study, we conducted the analysis in the STRING database [69].

### 4.7. Quantitative Real-Time PCR

After RNA isolation, as previously described, 1 µg of total RNA from three independent samples (*n* = 3) in each study group was reverse transcribed at 50 °C for 60 min using Superscript III reverse transcriptase (Invitrogen). 

Relative amounts of cDNA were quantified by quantitative real-time PCR using SYBR Green fluorophore (PowerUp SYBR Green Master Mix, Applied Biosystems, ThermoFisher Scientific, Waltham, MA, USA) and QuantStudio 3 system (Applied Biosystems) according to the manufacturer’s protocol. In all cases, 10 ng of cDNA were amplified using primers for the follow human target genes: *APP695* (forward: 5′-GACGATGAGGATGGTGATGA-3′; reverse: 5′-CTGGCTGCTGTTGTAGGAACT-3′), *GFAP* (forward: 5′-GTTCTTGAGGAAGATCCACGA-3′; reverse: 5′-CTTGGCCACGTCAAGCTC-3′), *TUBB3* (forward: 5′-GCAACTACGTGGGCGACT-3′; reverse: 5′-ATGGCTCGAGGCACGTACT-3′), *S100B* (forward: 5′-GGAAGGGGTGAGACAAGGA-3′; reverse: 5′-GGTGGAAAACGTCGATGAG-3′), and housekeeping gene *TBP* (forward: 5′-GAGCTGTGATGTGAAGTTTCC-3′; reverse: 5′-TCTGGGTTTGATCATTCTGTAG-3′). The QuantStudio 3 system was used to determine the amount of target mRNA in each sample, estimated by the 2^−ΔΔCt^ relative quantification method. Gene expression levels were normalized against human *TBP* gene levels in each sample.

### 4.8. Immunocytochemistry

hNS1 cells cultivated in multiwell plates (at 4 days after nucleofection) were fixed with PFA (4%, Sigma) for 10 min. Subsequently, cultures were blocked with normal horse serum (NHS) (5%, Gibco) and triton X-100 (0.25%, Merck, Darmstadt, Germany) in PBS for 30 min and incubated overnight at 4 °C with mouse antibodies against GFAP (glial fibrillary acid protein; 1:1000, BD Pharmigen, BD Biosciences, Madrid, Spain) and β-III-tubulin (class III β-tubulin; 1:3000, Biolegend, San Diego, CA, USA). Then, cultures were washed with triton X-100 in PBS and incubated with Alexa 555-conjugated antibody (donkey anti-mouse; 1:500, Invitrogen) for 1 h in darkness. All antibodies used were diluted in NHS (1%, Gibco) and triton X-100 in PBS. For determination of total cells, the cell nuclei were stained with Hoechst 33258 (5 μg/mL, Invitrogen) in PBS for 10 min. Visualization and analysis of preparations were performed using fluorescence microscopy (Leica DMIL LED, Leica Biosystems, Deer Park, IL, USA). Image analysis was performed using Photoshop CS6 after randomly capturing at least 8 separate fields per well, with a minimum of three wells per study group (*n* = 3).

### 4.9. Western Blot

hNS1 cells cultivated in multiwell plates were dissociated with trypsin and centrifuged in PBS. The pellets were treated with lysis buffer (RIPA, Cell Signaling Technology, Danvers, MA, USA) to obtain cell extracts. Then, 50 μg of total proteins from cell extracts were loaded on sodium dodecyl sulphate-polyacrylamide gels (10%, BioRad, Hercules, CA, USA), electrophoresed (SDS-PAGE), and transferred to nitrocellulose membranes (GE Healthcare, Madrid, Spain). Membranes were blocked with milk (5%) and Tween20 (0.05%, Sigma) in TBS for 1 h and incubated overnight at 4 °C with mouse antibodies against APP (clone 22C11; 1:1000, Millipore, Merck, Darmstadt; Germany) and β-actin (1:1000, Sigma). Then, membranes were washed with Tween20 in TBS and incubated with HRP-conjugated antibody (horse anti-mouse peroxidase; 1:3000, Vector Laboratories, Newark, CA, USA) for 1 h. All antibodies used were diluted in milk and Tween20 in TBS. Visualization of immunoreactive bands was performed using ECL chemiluminescent system (Millipore) according to the manufacturer’s instruction. For quantification of blot images, densitometry analyses were realized using the software ImageJ v.1.54a. Band intensity was measured as a ratio of the protein of interest (APP using 22C11 antibody) to β-actin from three independent experiments.

### 4.10. Statistical Analysis

Statistical tests were performed using GraphPad Prism 8. Results are shown as the mean ± SD of data from three independent samples (*n* = 3) in each study group. The statistical significance of data was determined using *T*-test (unpaired), and, in all cases, a *p*-value < 0.05 was considered to be statistically significant (* *p* < 0.05, ** *p* < 0.01, *** *p* < 0.001).

## Data Availability

The datasets generated during the current study are available in NCBI Gene Expression Omnibus (GEO) repository, accession number: GSE233651.

## Supplementary Materials

The supporting information can be downloaded at: https://www.mdpi.com/article/10.3390/ijms241612964/s1.
